# Screening of Metal Hypersensitivity in Pediatric Spine Surgery vs. Pectus Excavatum: A Comparative Cohort Study

**DOI:** 10.7759/cureus.93222

**Published:** 2025-09-25

**Authors:** Wasim Shihab, Alvin Jones, Scott Emmert, Raphael H Parrado, Tiffany Ruan, Nichole Leitsinger, Lindsay Schultz, Viral Jain, Michael Sherenian, Amal Assa'ad

**Affiliations:** 1 Orthopedic Surgery, Cincinnati Children's Hospital Medical Center, Cincinnati, USA; 2 Pediatric Critical Care, Cincinnati Children's Hospital Medical Center, Cincinnati, USA; 3 Allergy and Immunology, Cincinnati Children's Hospital Medical Center, Cincinnati, USA

**Keywords:** adolescent, metal allergy, metal hypersensitivity, nuss procedure, pectus excavatum, pediatric spine surgery, preop screening, spinal fusion

## Abstract

Introduction: Metal hypersensitivity is a type-IV delayed-type hypersensitivity reaction that may contribute to complications in orthopedic surgeries. While its clinical relevance remains uncertain, immune responses to implants have been documented. Pediatric spinal deformity correction and pectus excavatum repair often involve the use of metal implants that contain known allergens, including nickel, cobalt, and chromium. Despite extensive adult data, prevalence in pediatric spine surgery remains underexplored.

Methods: This retrospective cohort study reviewed pediatric patients who underwent posterior spinal fusion with instrumentation or the Nuss procedure for pectus excavatum repair between 2014 and 2020. The spine cohort underwent selective preoperative screening based on a self-reported metal sensitivity questionnaire, while the pectus cohort received routine preoperative Patch Metal Allergy Testing (PMAT). Statistical analyses, including Fisher’s Exact Test and logistic regression, were performed to compare hypersensitivity prevalence and associated risk factors. Odds ratios (OR) with 95% confidence intervals (CI) were calculated for categorical comparisons.

Results: Of 796 spinal fusion patients, 118 (14.8%) underwent PMAT, yielding a 3.3% hypersensitivity rate. In contrast, 815 of 864 pectus excavatum patients completed PMAT, yielding a 12.8% hypersensitivity rate. Patients in the spine cohort were older (median age 15 vs. 14 years; p < 0.01) and more likely to be female (64.2% vs. 22.7%; OR = 6.11, 95%CI: 4.91-7.60; p < 0.0001). Despite this, they were significantly less likely to test positive for metal hypersensitivity (OR = 0.23, 95%CI: 0.15-0.36; p < 0.0001). When viewed from the pectus cohort perspective, this corresponds to 4.33 times higher odds of PMAT positivity.

Conclusion: The lower prevalence in spine patients likely reflects differences in PMAT determination rather than true population differences. This study highlights the potential underdiagnosis of hypersensitivity in the spine cohort when using selective screening, compared to routine testing in the pectus cohort. Determining higher yield screening criteria for spine surgery may result in detection rates more comparable to those found in the pectus cohort. Further research is needed to refine preoperative screening protocols and evaluate the clinical impact of metal hypersensitivity in pediatric orthopedic surgery.

## Introduction

Metal sensitivity is a type-IV delayed-type hypersensitivity reaction linked to exposure to metal-containing implants. Though the exact clinical significance of metal hypersensitivity remains under debate, it is evident that some patients exhibit excessive eczematous immune reactions directly associated with implanted metallic materials [1].

Pediatric spinal deformity correction and pectus excavatum repair involve implanting devices composed of various metal alloys. These alloys may contain elements such as aluminum, nickel, molybdenum, carbon, titanium, cobalt, chromium, vanadium, or others [2,3]. Nickel, cobalt, and chromium have high rates of contact allergy, with nickel being the most common culprit in general populations, especially among female populations. This sensitivity is thought to be exposure-dependent, influenced by environmental metal exposure from items like jewelry, clothing fasteners, dental restorations, and mobile phones [4,5].

One study found positive patch tests in about 35% of women and 15% of men in the general Norwegian population, with nickel (17.6%) and cobalt (2.8%) being the main allergens [6]. Shah et al. reported that 6.4% of Nuss procedure patients had clinical or patch test evidence of metal allergy [7].

Research in orthopedic surgeries, notably in joint arthroplasty, demonstrates that metal hypersensitivity can complicate outcomes, including pain, erythema, delayed wound healing, and implant failure [1,8]. Park et al. showed that patients with osteolysis adjacent to implants had higher rates of cobalt allergy [9]. 

While research on metal hypersensitivity in spine surgery remains limited to a few case reports [10,11], a recent study by Emmert et al. (2025) using the same spine cohort analyzed in the present study provided one of the first systematic evaluations of this issue, reporting a 3.3% prevalence of hypersensitivity in pediatric patients undergoing spinal instrumentation [12]. Their study used a selective screening approach, where only patients with a self-reported history of metal sensitivity underwent Patch Metal Allergy Testing (PMAT). These findings highlight the potential underdiagnosis of hypersensitivity cases when relying solely on self-reported screening, a concern that we further explore by comparing this spine cohort to a pectus excavatum cohort that underwent routine PMAT testing [12].

Although metal hypersensitivity has been investigated in adult arthroplasty and pectus patients, no comparative study has addressed detection differences arising from screening protocols in pediatric populations. This study aimed to compare the prevalence of metal hypersensitivity between pediatric patients undergoing posterior spinal fusion and pectus excavatum repair, and to evaluate whether selective questionnaire-based screening in spine patients is as effective as routine patch testing in pectus patients.

## Materials and methods

This was a retrospective cohort study conducted at a single tertiary pediatric hospital, the Cincinnati Children's Hospital, Cincinnati, Ohio, United States. The study was approved by the Cincinnati Children's Hospital Institutional Review Board (IRB ID: 2023-0105).

Study population

The study population included pediatric patients under 18 years of age who underwent primary posterior spinal fusion with instrumentation at the study hospital between January 1, 2014, and December 31, 2020. Eligible patients were identified through institutional surgical databases and chart review. Spinal deformities were categorized based on etiology into idiopathic, neuromuscular, syndromic, congenital, or other causes, according to clinical and radiographic diagnoses at the time of surgery. A subsequent IRB-approved amendment permitted the inclusion of a comparison cohort consisting of pediatric patients who underwent the Nuss procedure for correction of pectus excavatum at the same institution during the same time frame. The inclusion criteria for both cohorts required the availability of complete preoperative screening data. Patients who underwent revision surgeries or failed to complete the metal hypersensitivity assessment were excluded from the final analysis.

Preoperative screening and metal hypersensitivity testing

In the spinal fusion cohort, all patients underwent a standardized preoperative screening process, which included a questionnaire to identify possible metal hypersensitivity with the question: “Has the patient ever developed a red, patchy rash after contact with metal buckles, snaps, zippers, or inexpensive jewelry?” Patients who responded affirmatively were referred to the institution’s Allergy and Immunology department for comprehensive PMAT. No patients who screened positive on the questionnaire declined PMAT testing.

PMAT was conducted by board-certified allergists using a panel of standardized allergens, including: nickel sulfate hexahydrate 5%, cobalt (II) sulfate 2.5%, manganese chloride 0.5%, copper sulfate hexahydrate 2%, aluminum hydroxide 10%, titanium (IV) oxide 0.1%, ammonium heptamolybdate 1%, vanadium pentoxide 10%, chromium (III) chloride 1%.

A positive result was defined as a grade 2+ reaction or greater, consistent with a clinically significant contact hypersensitivity reaction. In cases of confirmed hypersensitivity, implant materials were selected to avoid the identified allergens, based on the guidance of the surgical and allergy teams.

In contrast, all patients in the pectus excavatum cohort underwent routine pre-operative PMAT testing regardless of questionnaire responses, as part of a standardized institutional protocol initiated during the study period. For both cohorts, if a patient underwent more than one procedure, data were consolidated to represent the initial surgical episode. Patients were excluded if instrumentation was not performed or if they did not complete PMAT testing.

Statistical analysis

Categorical variables were compared using Fisher’s Exact Test or Chi-Square Test, as appropriate. Continuous variables such as age were analyzed using the Mann-Whitney U test due to non-normal distribution. Odds ratios (ORs) with 95% confidence intervals (CIs) were calculated to assess the strength of association between group membership and outcomes such as gender distribution and PMAT positivity. Statistical significance was defined as a two-tailed p-value < 0.05. All analyses were conducted using GraphPad Prism (Dotmatics, Boston, Massachusetts, United States).

## Results

**Figure 1:**
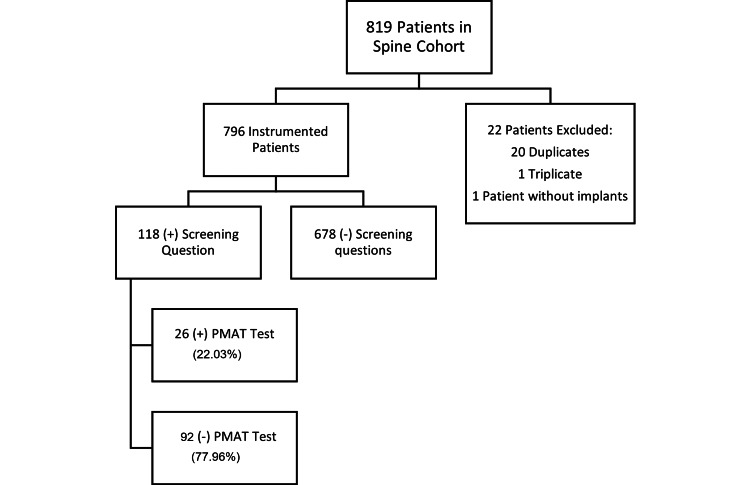
Flowchart for the spinal cohort screening PMAT: Patch Metal Allergy Testing

**Figure 2:**
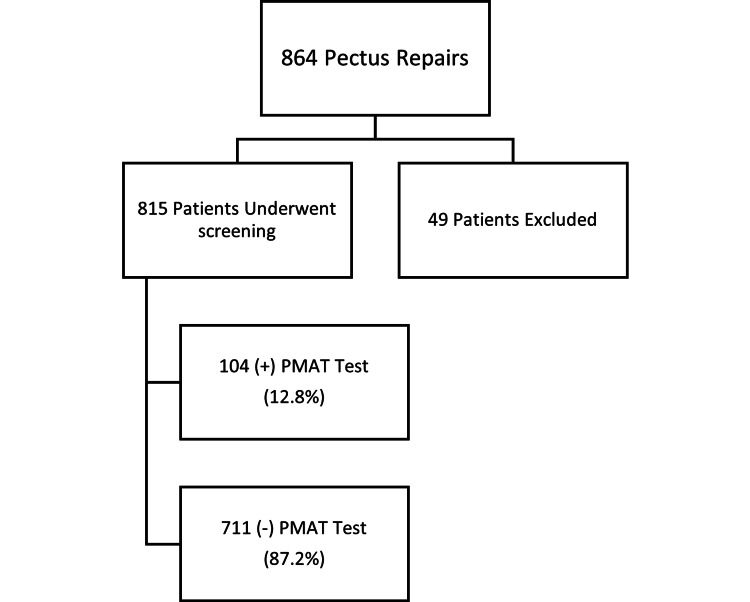
Flowchart for the pectus excavatum cohort screening PMAT: Patch Metal Allergy Testing

A total of 796 pediatric patients underwent posterior spinal fusion with instrumentation during the study period. Of these, 118 patients (14.8%) responded affirmatively to a preoperative screening question regarding metal sensitivity and were subsequently referred for PMAT (Figure 1). The pectus cohort comprised 864 patients who underwent surgical repair for pectus excavatum, of whom 815 (94.3%) completed routine PMAT testing. Forty-nine patients in the pectus group were excluded due to incomplete testing (Figure 2).

Patients in the spine cohort were significantly older (median age 15 vs. 14 years; p < 0.01, Mann-Whitney U test) and had a significantly higher proportion of female patients compared to the pectus cohort (64.2% vs. 22.7%; OR = 6.11, 95%CI: 4.91-7.60; p < 0.0001) (Table 1). Among the spine patients who screened positive on the questionnaire, the distribution of spinal deformity etiology included idiopathic (82%), neuromuscular (8%), syndromic (5%), congenital (4%), and other causes (1%). 

**Table 1:** Demographic and implant comparisons between cohorts

Variable	Scoliosis cohort	Pectus cohort	p-value	OR (95% CI)
Number of patients	796	815		
Median age (years)	15	14	<0.01	
Female sex, n (%)	511 (64.2%)	185 (22.7%)	<0.0001	6.11 (4.91–7.60)
Implanted metal, n (%)				
Titanium	300 (37.7%)	291 (35.7%)	0.44	1.09 (0.89–1.33)
Cobalt Chromium	457 (57.4%)	0 (0%)	<0.0001	2197.9 (136.9–35293.7)
Stainless Steel	32 (4.0%)	573 (70.3%)	<0.0001	0.018 (0.012–0.026)
Mixed Titanium/Cobalt Chromium	7 (0.9%)	0 (0%)	0.007	15.5 (0.88–271.7)

The overall prevalence of positive PMAT results was 3.3% (26/796) in the spine cohort and 12.8% (104/815) in the pectus cohort, yielding a statistically significant difference (OR = 0.23, 95%CI: 0.15-0.36; p < 0.0001). When viewed from the pectus perspective, this corresponds to 4.33 times higher odds of a positive PMAT result in pectus patients compared to spine patients (Table 2). Nickel was the most frequently identified allergen in both cohorts, accounting for 77% of positive PMAT results in the spine group and 74% in the pectus group. Other metals identified in positive tests included cobalt (35% spine vs. 42% pectus), manganese (12% vs. 7%), and copper (4% vs. 0%). Cobalt chromium was the predominant implant alloy utilized in the spine cohort (39%), whereas stainless steel was most used in the pectus cohort (66%).

**Table 2:** PMAT results for both cohorts PMAT: Patch Metal Allergy Testing

Variable	Spine (n=796), n (%)	Pectus (n=815), n (%)	P-value	OR (95% CI)
History of Reaction to Metals	118 (14.8)	285 (35)		
Positive PMAT Test (%)	26 (3.3)	104 (12.8)	<0.0001	0.23 (0.15–0.36)
Nickel Sulfate Hexahydrate 5%	20 (76.9)	77 (74)	1.00	1.17 (0.42–3.22)
Cobalt (II) Sulphate 2.5%	9 (34.6)	45 (43)	0.51	0.69 (0.28–1.70)
Manganese Chloride 0.5%	3 (11.5)	7 (6.7)	0.42	1.81 (0.43–7.53)
Copper Sulfate Hexahydrate 2%	1 (3.8)	0	0.20	12.3 (0.49–310.7)
Aluminum Hydroxide 10%	0	0	1.00	3.94 (0.08–203.4)
Titanium (IV) Oxide 0.1%	0	0	1.00	3.94 (0.08–203.4)
Ammonium Hepatomolybdate 1%	0	0	1.00	3.94 (0.08–203.4)
Vanadium Pentoxide 10%	0	4 (3.8)	0.58	0.42 (0.02–8.07)
Chromium (III) Chloride 1%	0	2 (1.9)	1.00	0.77 (0.04–16.6)

## Discussion

This study is the first to systematically examine the prevalence of metal hypersensitivity in pediatric spine surgery patients compared to a pectus excavatum cohort. The findings suggest that the prevalence of metal hypersensitivity is significantly lower in the spine cohort, potentially due to the selective nature of its preoperative screening process, which relies on a self-reported history of contact dermatitis. In contrast, the routine PMAT testing in the pectus cohort likely captured more cases of hypersensitivity.

Female individuals generally have a higher prevalence of metal hypersensitivity, likely due to earlier exposure to nickel through jewelry and ear piercings. Thyssen et al. suggested that 17% and 3% of women and men, respectively, have hypersensitivity to nickel, and it is directly related to exposure [4,13]. The predominance of female patients in the spine cohort, coupled with their older average age, would suggest a higher prevalence of hypersensitivity. However, the selective screening process may have contributed to underestimating the true prevalence in this group. These findings advocate for broader screening criteria in high-risk spine patients, especially given the low burden of testing indicated by the NNT. Nevertheless, this only accounts for the nickel exposure and does not account for the cobalt hypersensitivity, which was found to be the second most prevalent. Cobalt is not a metal frequently encountered in the general population, so the development of this hypersensitivity reaction is not well understood.

The screening questionnaire used in the spine cohort, while selective, demonstrated a PPV of 22.0%, with an NNT of 4.6. This indicates that it is quite efficient and supports its clinical utility in settings where universal PMAT is not feasible. These findings emphasize the importance of balancing resource allocation with diagnostic accuracy in preoperative planning.

While the questionnaire was administered selectively to spine patients, its performance offers insight into its practical application. The modest positive predictive value suggests that the majority of those who report a history of metal sensitivity may not have a true hypersensitivity when tested formally. However, the number needed to test, just under five patients to identify one with a confirmed metal allergy, supports its role as a reasonable triage tool in the preoperative setting. In institutions where universal PMAT is impractical, a targeted screening approach guided by a simple questionnaire may still provide clinical value when embedded within a broader risk-stratification framework.

Not all patients with metal hypersensitivity exhibit symptoms; metal hypersensitivity prevalence in the general population is around 10-15% [1], but symptomatic metal hypersensitivity in total joint arthroplasty is estimated to be less than 1% [14]. Niki et al. found a significant association between cobalt chromium sensitivity and the development of eczema, where they offered cobalt chromium sensitivity as a potential predictor for symptomatic metal hypersensitivity [15].

Metal hypersensitivity can mimic postoperative infections with symptoms such as increased pain, erythema, and delayed wound healing, making diagnosis challenging [1]. Misdiagnosis can lead to unnecessary interventions, including prolonged antibiotic therapy or revision surgery. Routine preoperative PMAT testing, as implemented in the pectus cohort, may provide a more comprehensive approach to identifying at-risk patients, and this has been recommended by some authors studying the role metal implants play in the development of hypersensitivity [5]. Selecting hypoallergenic implant materials for patients with known hypersensitivity could mitigate complications and improve outcomes [16]. Nevertheless, a Delphi study of surgeons performing joint arthroplasty in the United Kingdom showed most do not screen for metal hypersensitivity and continue to use traditional cobalt chromium/stainless steel implants regardless of the patient’s metal allergy status [17].

The discrepancy in metal hypersensitivity prevalence seen between these two cohorts highlights the need for a more robust screening tool in the spine cohort. Obermeyer et al. suggested incorporating family and personal history and risk factors like female gender into the pre-operative screening [16]. Incorporating demographic risk factors, family history, and routine PMAT testing could reduce the risk of false negatives and better identify patients at risk of hypersensitivity reactions. Expanding the criteria for testing may also help guide implant material selection and reduce preventable complications.

This study is limited by its retrospective design and reliance on selective screening based on patient-reported history, which likely underestimates the true prevalence of metal hypersensitivity in the spine cohort. The selective nature of questionnaire-based referral may also have introduced referral bias, as patients with more apparent or self-reported symptoms were more likely to undergo PMAT testing, potentially missing asymptomatic cases. Additionally, we did not directly assess clinical outcomes or postoperative symptoms in patients with confirmed hypersensitivity, and the absence of long-term follow-up further limits our ability to determine the clinical significance of hypersensitivity or its eventual impact on implant performance. Because hypersensitivity reactions can mimic other postoperative complications, such as increased pain or delayed wound healing, future prospective studies with standardized screening protocols and extended follow-up are needed to better distinguish true hypersensitivity from other causes of postoperative morbidity and to evaluate its long-term implications.

## Conclusions

Preoperative screening for metal hypersensitivity is an important consideration in pediatric surgical planning. In this study, the prevalence of hypersensitivity appeared lower in the spine cohort compared to the pectus cohort; however, this difference likely reflects the use of selective, questionnaire-based screening in the spine group versus routine PMAT testing in the pectus group. These methodological differences limit direct comparison but also highlight the practical trade-off between selective and universal screening. Even with its limitations, a simple questionnaire proved to be quite efficient, identifying children at risk in settings where universal PMAT is not feasible. Refining such targeted tools by incorporating broader risk factors may further improve accuracy and support safer, more personalized implant selection.
